# Supporting countries to ensure the continuum of integrated care for older people

**DOI:** 10.1007/s40520-025-02970-4

**Published:** 2025-03-20

**Authors:** Yuka Sumi, Rachel Albone, Matteo Cesari

**Affiliations:** Ageing and Health Unit, Department of Maternal Newborn, Child and Adolescent Health and Ageing, World Health Organization, Avenue Appia 20, Geneva, 1202 Switzerland

The United Nations Decade of Healthy Ageing (2021–2030) is a global collaboration aimed at improving the lives of older people, their families, and the communities in which they live [1]. Under this framework, coordinated by the World Health Organization (WHO), specific work supports that older people can access a continuum of integrated care responsive to their diverse needs, priorities, and values [2, 3]. For this purpose, strengthening primary health care (PHC) systems, effective collaboration and coordination among care providers, and adopting a person-centred approach focused on maintaining functional ability are required. Integrated care can help promote a better allocation of resources, reduce healthcare costs, and improve health outcomes for the individual, in turn contributing to the sustainability of healthcare systems and society’s well-being [4]. The transformation of health systems to enable the delivery of a continuum of integrated care is particularly critical for low- and middle-income countries, which are experiencing an exponential and rapid ageing of their populations while facing challenges in addressing basic care needs [5, 6].

The integrated care for older people approach, referred with the acronym “ICOPE”, is the WHO’s approach to provide a continuum of integrated care that helps to reorient health and social services towards more person-centred and coordinated care. It focuses on the optimisation intrinsic capacity and functional ability in older people. In January 2025, the WHO launched the second edition of *Integrated Care for Older People: Guidance for Person-Centred Assessment and Pathways in Primary Care* (ICOPE handbook 2nd edition) [7]. The document emphasises the importance of delivering personalised, comprehensive, coordinated care to older people, supporting a shift from traditional disease-oriented approach towards more holistic care. It offers evidence-based interventions for health and care workers in primary care including community, drawing on the valuable experiences gained from implementing ICOPE across various settings, countries, and regions, as well as integrating the latest WHO guidance on related health topics.

The ICOPE handbook 2nd edition introduces several key changes to enhance the care pathway for older people, evolving from the previous version. In particular, it promotes the integration of more accessible tools and methodologies to facilitate implementation and dissemination regardless of resource constraints.

In the second edition, the ICOPE care pathway in primary care has been streamlined from five to four “steps” probably representing the most visible change from the previous version. The ICOPE approach now consists of (1) basic assessment and community-level interventions, (2) in-depth assessment, (3) development of a personalised care plan, and (4) implementation and monitoring. The role of and support for community stakeholders and carers is now mainstreamed throughout the different steps (rather than only being included as part of a standalone step to facilitate implementation of the care plan).

The first step of the ICOPE care pathway in primary care is now called “Basic assessment and initial intervention” (in the previous version: “Screening”). This change reflects the expansion of the first step and a change to the services and support being provided in the community. The spectrum of the basic assessment is now broader than before, additionally focusing on the presence of social issues together with intrinsic capacity impairments. Furthermore, the basic assessment, likely to be conducted by a community health worker or non-clinical personnel, is now linked to the delivery of immediate and cost-effective interventions, including provision of health and lifestyle advice, promotion of vaccinations, connection to available care and support services and referral to primary care.

The ICOPE handbook 2nd edition proactively encourages the use of preventive strategies, including through increased community engagement. Examples of how community stakeholders (such as individuals, non-governmental organisations, senior citizen clubs, peer support groups, and volunteers) can help enhance access to care and empower older people in making health-related decisions are now provided in the document.

Increased attention is given to the need for social support and the role of carers. Specific questions aimed at better understanding the social context and the carers situation are included in the basic assessment, allowing the immediate identification of risks and needs that may hamper the overall case management. Such a comprehensive person-centred approach at the first contact with an older person facilitates bridging health and social services, potentially expanding opportunities to identify those who need care and offer multisectoral solutions. It is also recognised that carers, who may also be care recipients, require specific training and support. Strategies are included that can improve the quality of care they offer, help prevent burnout, and promote preventive strategies for their well-being.

The ICOPE handbook 2nd edition includes an additional care pathway targeting urinary incontinence. Despite evidence-based interventions available [4], urinary incontinence still represents a highly prevalent [8], underreported [9], and burdensome condition in older people [10, 11]. Encouraging a proactive approach to urinary incontinence by health and care workers may open opportunities for care, frequently hampered by the associated stigma and embarrassment [12].

Finally, it is important to highlight how the present document is clearly placed to ensure the delivery of a continuum of integrated care for older people that is being advocated for [3]. In fact, it considers the full spectrum of care, from health promotion and prevention, treatment, palliative care to long-term care and support to those who require assistance from others in their daily lives. Indeed, it is not sufficient to simply integrate services and generate a multidisciplinary environment; it is also critical to simultaneously establish a longitudinal process of monitoring and revision of care plans to adapt strategies according to the evolving needs and priorities of the older person.

As a concluding remark, it is impossible not to mention how ICOPE is consistent with and based on the large body of evidence built by geriatricians over the past decades. Comprehensive assessment, focus on capacities and abilities, multidisciplinary and multisectoral work, personalisation of care, and attention to the social context and carers’ roles and needs are just a few examples linking ICOPE to the basic principles of geriatric medicine [13–17]. The ICOPE handbook 2nd edition may bring an opportunity to implement and disseminate good clinical practice, especially in settings and countries where the number of older people is increasing but geriatric care is not yet sufficiently established. In this context, health and care workers with competencies in geriatric care must collaborate with community stakeholders, acting as both capacity builders and care providers.
